# Phenolic Profiles and Antioxidant Activities of 30 Tea Infusions from Green, Black, Oolong, White, Yellow and Dark Teas

**DOI:** 10.3390/antiox8070215

**Published:** 2019-07-10

**Authors:** Cai-Ning Zhao, Guo-Yi Tang, Shi-Yu Cao, Xiao-Yu Xu, Ren-You Gan, Qing Liu, Qian-Qian Mao, Ao Shang, Hua-Bin Li

**Affiliations:** 1 Guangdong Provincial Key Laboratory of Food, Nutrition and Health, Department of Nutrition, School of Public Health, Sun Yat-Sen University, Guangzhou 510080, China; 2 Department of Food Science & Technology, School of Agriculture and Biology, Shanghai Jiao Tong University, Shanghai 200240, China; 3 Institute of Urban Agriculture, Chinese Academy of Agricultural Sciences, Chengdu 610213, China

**Keywords:** tea, *Camellia sinensis*, antioxidant activity, polyphenol, catechin, caffeine

## Abstract

Tea is among the most consumed drink worldwide, and its strong antioxidant activity is considered as the main contributor to several health benefits, such as cardiovascular protection and anticancer effect. In this study, the antioxidant activities of 30 tea infusions, which were obtained by the mimic of drinking tea of the public, from green, black, oolong, white, yellow and dark teas, were evaluated using ferric-reducing antioxidant power (FRAP) and Trolox equivalent antioxidant capacity (TEAC) assays, ranging from 504.80 ± 17.44 to 4647.47 ± 57.87 µmol Fe^2+^/g dry weight (DW) and 166.29 ± 24.48 to 2532.41 ± 50.18 µmol Trolox/g DW, respectively. Moreover, their total phenolic contents (TPC) were detected by Folin-Ciocalteu assay and were in the range of 24.77 ± 2.02 to 252.65 ± 4.74 mg gallic acid equivalent (GAE)/g DW. Generally, Dianqing Tea, Lushan Yunwu Tea, and Xihu Longjing Tea showed the strongest antioxidant activities among 30 teas. Furthermore, the phenolic compounds in tea infusions were identified and quantified, with catechins most commonly detected, especially in green tea infusions, which were main contributors to their antioxidant activities. Besides tea polyphenols, considerable content of caffeine also presented in 30 tea infusions.

## 1. Introduction

Tea (*Camellia sinensis*) is the most consumed drink in the world except for water, owing to its pleasant sensory properties, broad health benefits and unique sociocultural characteristics [1]. Usually, tea could be classified into six categories, i.e., green tea (unfermented), yellow tea (slight-fermented), white tea (mild-fermented), oolong tea (semi-fermented), black tea (fermented) and dark tea (post-fermented) according to the varying fermentation extent [2]. Accumulating clinical research has indicated the preventive and therapeutic effects of tea on oxidative stress-related diseases, like cardiovascular disease, cancer, liver disease, type 2 diabetes and cognitive dysfunction [3,4,5,6,7,8]. Moreover, several studies have shown that polyphenols and caffeine are the main ingredients responsible for the various bioactivities and distinctive sensory properties of teas [2,9,10,11,12,13]. The most favorable antioxidant activities of tea infusions are attributed to polyphenols greatly [14,15,16]. The in vivo antioxidant activities of tea polyphenols cannot be simply extrapolated from their in vitro antioxidant effects due to their differences in bioavailability [17]. On the one hand, some polyphenols with strong in vitro antioxidant activities cannot pass the small intestinal barrier due to their polarity, solubility, or high molecular weight, and will possess weak in vivo antioxidant activities. On the other hand, some polyphenols with weak in vitro antioxidant activities can contribute to strong in vivo antioxidant activities after being transformed by intestinal enzymes or metabolized by gut microbiota [18]. In addition, the in vitro and in vivo antioxidant activities of some natural products could be consistent in certain conditions. Overall, in vitro antioxidant activity and phenolic profiles of tea can provide important references for the further study of in vivo antioxidant effects.

In a previous paper, the antioxidant polyphenols in different fractions of 30 teas, including fat-soluble, water-soluble, and insoluble-bound fractions, were evaluated after extraction by different organic solvents [19]. Using this method, it was expected that the antioxidants in tea could be completely extracted, and the total antioxidant capacity of tea could be obtained. However, this extraction method is very different compared to the habit of the public for tea drinking, where only water is used.

In this paper, therefore, we aimed to simulate the way of tea drinking to extract antioxidants in teas. The antioxidant activities of aqueous extracts (infusions) of 30 well-known teas from China, including 4 black teas, 5 dark teas, 9 green teas, 4 oolong teas, 3 white teas, and 5 yellow teas were evaluated using ferric-reducing antioxidant power (FRAP) and Trolox equivalent antioxidant capacity (TEAC) assays, and their total phenolic contents (TPC) were determined by the Folin–Ciocalteu method. Moreover, polyphenols and caffeine in 30 tea infusions were detected using high-performance liquid chromatography coupled with photodiode array detector (HPLC-PDAD). Tea infusions could have different types and contents of antioxidants compared to the fat-soluble, water-soluble, and insoluble-bound fractions of tea. The results obtained can provide guidance for the public to select tea with strong antioxidant capacity. The results are also very helpful for the nutritionist and epidemic experts to calculate/assess the intake amounts of antioxidants from tea.

## 2. Materials and Methods

### 2.1. Chemicals

The 2,4,6-tri(2-pyridyl)-s-triazine (TPTZ), 2,2′-azino-bis(3-ethylbenothiazoline-6-sulphonic acid) diammonium salt (ABTS), 6-hydroxy-2,5,7,8-tetramethylchromane-2-carboxylic acid (Trolox) and Folin and Ciocalteu’s phenol were produced by Sigma-Aldrich (Saint Louis, MO, USA). The standard chemicals, i.e., gallocatechin (GC), epigallocatechin (EGC), epigallocatechin gallate (EGCG), catechin (C), epicatechin (EC), epicatechin gallate (ECG), catechin gallate (CG), gallocatechin gallate (GCG), gallic acid, chlorogenic acid, ellagic acid, myricetin, quercitrin, kaempferol, astragalin, quercetin, theaflavin and caffeine, were obtained from Derick Biotechnology Co., Ltd. (Chengdu, China). The methanol and formic acid were of chromatography-grade and purchased from Kermel Chemical Factory (Tianjin, China). All the other reagents (such as sodium carbonate anhydrous) were of analytical grade and obtained from Damao Reagent Factory (Tianjin, China).

### 2.2. Sample Preparation

The 30 teas (Table 1) were purchased from China. The sample (1 g) was extracted by 10 mL boiling distilled water in a 98 °C water bath shaker for 5 min, and the extraction procedures were repeated six times. All infusions were combined, and their antioxidant activities were evaluated immediately.

### 2.3. Detection of Antioxidant Capacity and Total Phenolic Content (TPC)

The FRAP assay was utilized to determine the reducing ability of tea infusions based on literature previously published with minor modification [20]. In brief, the properly diluted sample of 100 μL was mixed with 3 mL of FRAP reagent (a mixture of 300 mmol/L sodium acetate-acetic acid buffer, 10 mmol/L TPTZ solution, and 20 mmol/L ferric chloride solution at a volume ratio of 10:1:1). The reaction was conducted at room temperature for 4 min, and the absorbance was recorded at 593 nm. FeSO_4_ was used as a standard, and the results were expressed as µmol Fe^2+^/g dry weight (DW).

The TEAC assay was used to measure the free radical-scavenging ability of tea infusions based on previously published literature that was slightly modified [21]. Briefly, 0.1 mL of appropriately diluted sample was mixed with 3.8 mL of ABTS•^+^ working solution with absorbance controlled at 0.710 ± 0.050. After the reaction at room temperature for 6 min, the absorbance was measured at the wavelength of 734 nm. Trolox was used as a standard, and the results were presented as µmol Trolox/g DW.

The TPC was measured by the Folin–Ciocalteu method [22]. The 0.5 mL of properly diluted sample was mixed with 2.5 mL of 0.2 mol/L Folin-Ciocalteu reagent. After 4 min of reaction in dark at room temperature, 2 mL of saturated sodium carbonate solution (75 g/L) was added, and the reaction continued for 2 h in dark at room temperature. The absorbance was measured at a wavelength of 760 nm. Gallic acid was used as a standard, and the results were expressed as mg gallic acid equivalent (GAE)/g DW.

### 2.4. High-Performance Liquid Chromatography (HPLC) Analysis

Tea infusions were subjected to HPLC coupled with a PDAD (Waters, Milford, MA, USA) and an Agilent Zorbax Extend-C18 column (4.6 × 250 mm, 5 µm) for determination of phenolic compounds and caffeine. The HPLC method referred to previous literature with minor modification [23]. In brief, the mobile phase A and B were methanol and 0.1% formic (*v/v*), respectively, and the temperature was 35 °C with the flow rate of 1.0 mL/min. The elution gradient was set as follows: 5% A (0 min), 20% A (10 min), 22% A (15 min), 25% A (20 min), 40% A (40 min), 42% A (50 min), 50% A (60 min), 95% A (70 min), 5% A (70.1 min) and 5% A (75 min). Phenolic compounds and caffeine in tea infusions were identified via comparing their retention time and ultraviolet-visible (UV-vis) spectra with those of standard compounds. Quantitative analysis was performed according to the peak area under the maximal absorbance wavelength, and the contents were expressed as mg/g DW. The limit of detection (LOD) and limit of quantity (LOQ) of this method were 2 and 5 µg/mL, respectively.

### 2.5. Statistical Analysis

All data obtained after 3 repetitions were analyzed using SPSS 20.0. (IBM SPSS Statistics, IBM Corp, Somers, NY, USA) and expressed as mean ± standard deviation (SD). Significant differences among groups were manifested using one-way analysis of variance (ANOVA) followed by an LSD post hoc test, with a significant level set at 0.05. For potential correlation among measurements, the linear regression analysis was performed. Statistical significance was defined at *p* < 0.05.

## 3. Results

### 3.1. Antioxidant Capacities of Tea Infusions

**Figure 1:**
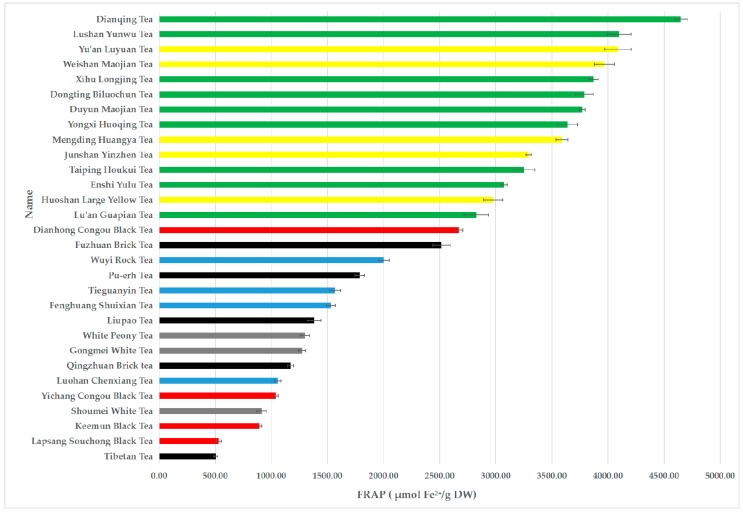
The FRAP values of 30 teas.

The FRAP values of different tea infusions were quite different and varied from 504.80 ± 17.44 to 4647.47 ± 57.87 µmol Fe^2+^/g DW (Table 1 and Figure 1). Among the 30 teas, the top 5 with the highest FRAP values were Dianqing Tea, Lushan Yunwu Tea, Yuan’an Luyuan Tea, Weishan Maojian Tea, and Xihu Longjing Tea, and their FRAP values were 4647.47 ± 57.87, 4099.47 ± 105.10, 4088.80 ± 118.39, 3967.47 ± 87.76 and 3872.80 ± 38.16 µmol Fe^2+^/g DW, respectively. The FRAP values of different categories of teas were in a decreasing order: green tea (3663.32 ± 535.63 µmol Fe^2+^/g DW), yellow tea (3582.93 ± 433.94 µmol Fe^2+^/g DW), oolong tea (1539.13 ± 351.86 µmol Fe^2+^/g DW), dark tea (1472.27 ± 691.91 µmol Fe^2+^/g DW), black tea (1283.47 ± 858.62 µmol Fe^2+^/g DW) and white tea (1160.80 ± 190.32 µmol Fe^2+^/g DW).

**Figure 2:**
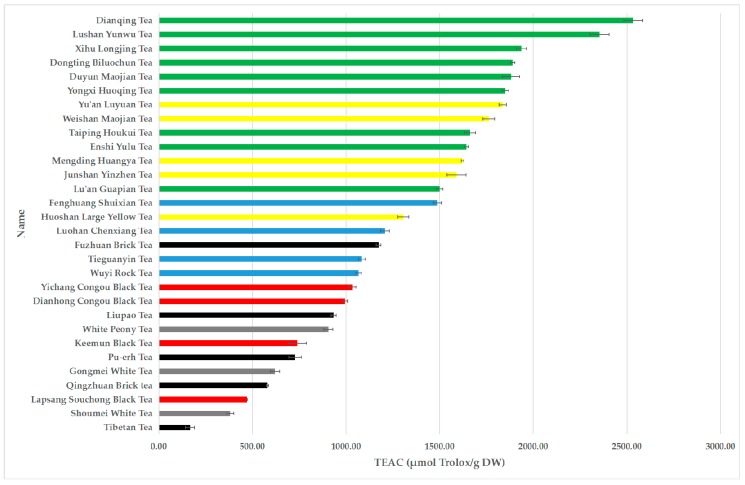
The TEAC values of 30 teas.

The TEAC values of different tea infusions varied greatly in a range of 166.29 ± 24.48 to 2532.41 ± 50.18 µmol Trolox/g DW (Table 1 and Figure 2). Among 30 teas, 5 green teas, i.e., Dianqing Tea, Lushan Yunwu Tea, Xihu Longjing Tea, Dongting Biluochun Tea, and Duyun Maojian Tea possessed the highest TEAC values of 2532.41 ± 50.18, 2353.21 ± 50.68, 1935.89 ± 26.32, 1889.22 ± 12.33 and 1880.54 ± 45.15 µmol Trolox/g DW, respectively. The TEAC values of different categories of teas decreased in the following order: green tea (1899.19 ± 315.79 µmol Trolox/g DW), yellow tea (1622.77 ± 190.92 µmol Trolox/g DW), oolong tea (1211.28 ± 176.81 µmol Trolox/g DW), black tea (809.97 ± 237.04 µmol Trolox/g DW), dark tea (715.99 ± 352.02 µmol Trolox/g DW) and white tea (635.42 ± 227.85 µmol Trolox/g DW).

### 3.2. Total Phenolic Content of Tea Infusions

**Figure 3:**
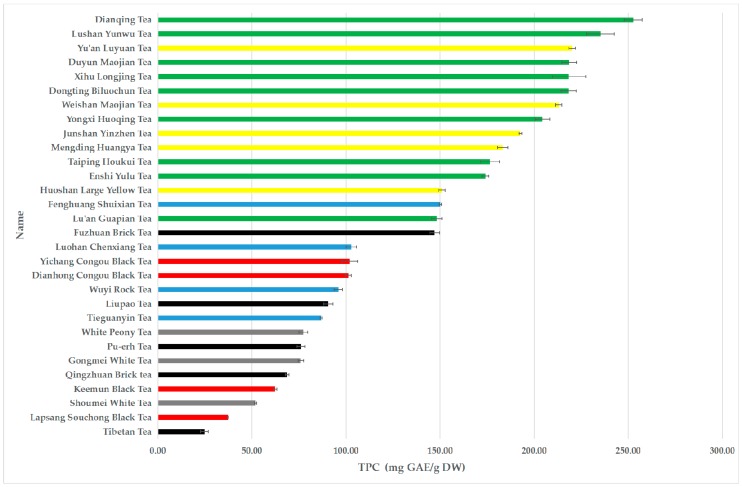
The TPC values of 30 teas.

The total phenolic contents of 30 tea infusions were quite differed ranging from 24.77 ± 2.02 to 252.65 ± 4.74 mg GAE/g DW (Table 1 and Figure 3). The phenolic contents of top 5 teas, i.e., Dianqing Tea, Lushan Yunwu Tea, Yuan’an Luyuan Tea, Duyun Maojian Tea, and Xihu Longjing Tea, were 252.65 ± 4.74, 235.27 ± 7.28, 220.08 ± 1.75, 218.55 ± 3.90 and 218.46 ± 8.82 mg GAE/g DW, respectively. The decreasing order of total phenolic contents of different categories of teas was green tea (205.16 ± 32.02 mg GAE/g DW), yellow tea (192.02 ± 25.36 mg GAE/g DW), oolong tea (108.91 ± 25.59 mg GAE/g DW), dark tea (81.43 ± 40.92 mg GAE/g DW), black tea (75.66 ± 28.70 mg GAE/g DW) and white tea (68.38 ± 12.44 mg GAE/g DW).

### 3.3. Correlations between Values of Ferric-Reducing Antioxidant Power (FRAP), Trolox Equivalent Antioxidant Capacity (TEAC), and TPC

**Figure 4:**
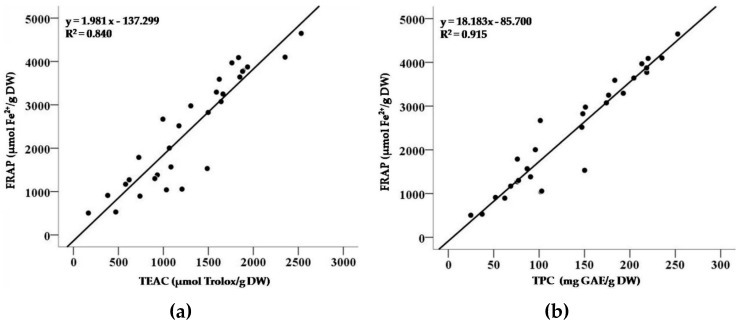

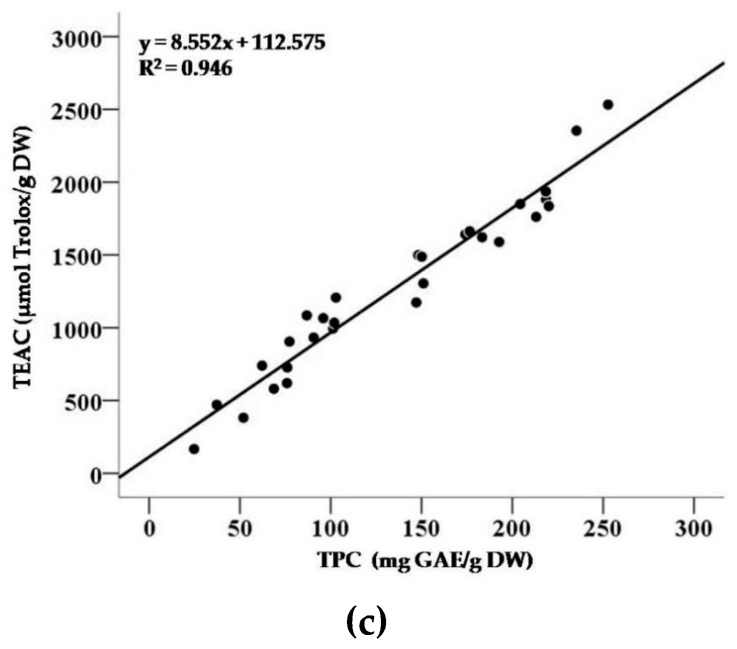
The correlations between values of FRAP and TEAC (**a**), FRAP and TPC (**b**), TEAC and TPC (**c**) of 30 teas.

The correlations between values of FRAP, TEAC, and TPC of 30 teas are presented in Figure 4. There was a strong correlation between FRAP and TEAC values of 30 teas, with *R^2^* of 0.840 (*p* < 0.001), which indicated that components in tea infusions possessed both good reducing ability and free radicals cleaning capacity. Moreover, the antioxidant activities of 30 teas were strongly correlated with their total phenolic contents (FRAP vs. TPC, *R*^2^ = 0.915, *p* < 0.001; TEAC vs. TPC, *R*^2^ = 0.946, *p* < 0.001). Thus, the phenolic compounds could play key roles in the strong antioxidant activities of teas.

### 3.4. Phenolic Profiles of Tea Infusions

**Figure 5:**
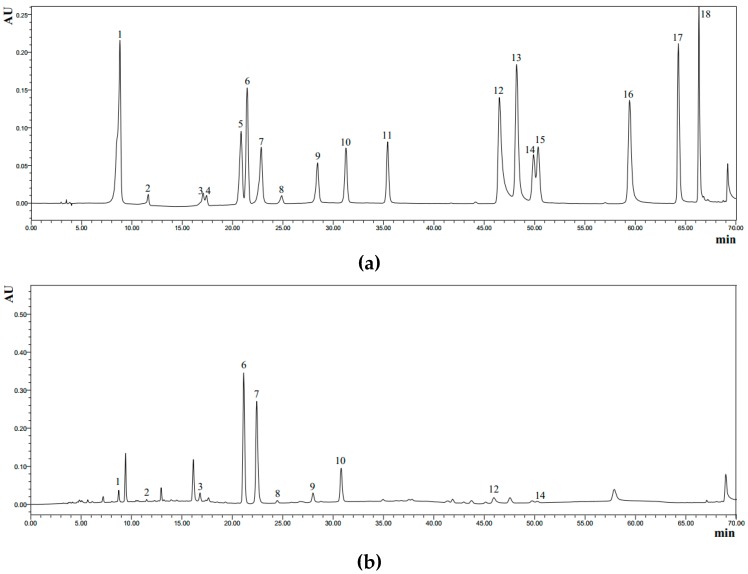
Chromatograms of standard chemicals (**a**) and Duyun Maojian Tea (**b**) under wavelength of 254 nm. 1, gallic acid; 2, gallocatechin; 3, epigallocatechin; 4, catechin; 5, chlorogenic acid; 6, caffeine; 7, epigallocatechin gallate; 8, epicatechin; 9, gallocatechin gallate; 10, epicatechin gallate; 11, catechin gallate; 12, ellagic acid; 13, myricetin; 14, quercitrin; 15, astragalin; 16, quercetin; 17, theaflavin; 18, kaempferol.

The phenolic compounds in different tea infusions were quite diverse (Table 2). The chromatograms of the standard compounds and Duyun Maojian Tea under the wavelength of 254 nm are presented in Figure 5. A total of 16 phenolic compounds were detected in infusions of 30 teas, including 8 catechins (GC, EGC, EGCG, C, EC, ECG, CG and GCG), 3 phenolic acids (gallic acid, chlorogenic acid and ellagic acid), 2 flavonols (quercetin and kaempferol), 2 flavonol glycosides (astragalin and quercitrin) and theaflavin. Catechins were the main phenolic compounds in tea infusions, accounting for 11.08%–16.21% dry weight of tea leaves. Specifically, EC (27 teas), EGCG (26 teas), ECG (24 teas), EGC (22 teas), GC (21 teas) and GCG (18 teas) could be detected in majorities of teas. Moreover, CG (7 teas) and C (6 teas) were detected in minorities of teas.

The highest contents of catechins were found in green tea infusions among 6 tea categories, and all green tea infusions possessed such 6 principal catechins as follows (with contents in a decreasing order): EGCG (48.48 ± 9.16 mg/g DW), EGC (45.25 ± 25.87 mg/g DW), ECG (20.47 ± 8.38 mg/g DW), EC (7.97 ± 1.46 mg/g DW), GC (6.19 ± 2.02 mg/g DW), GCG (6.09 ± 1.47 mg/g DW). Thus, EGCG was the richest catechins in green tea infusions.

The gallic acid was found in all 30 tea infusions in low contents varying from 0.29 ± 0.02 to 3.77 ± 0.32 mg/g DW. The top 5 teas with the highest contents of gallic acid were Yichang Congou Black Tea (3.77 ± 0.32 mg/g DW), Fuzhuan Brick Tea (3.22 ± 0.22 mg/g DW), Huoshan Large Yellow Tea (3.16 ± 0.26 mg/g DW), Fenghuang Shuixian Tea (3.05 ± 0.14 mg/g DW) and Keemun Black Tea (2.87 ± 0.15 mg/g DW). Furthermore, many tea infusions contained certain amounts of ellagic acid, quercitrin, astragalin, and chlorogenic acid, but the quercetin and kaempferol could only be found in 2 or 3 tea infusions. Additionally, theaflavin was only detected in Dianhong Congou Black Tea and Yichang Congou Black Tea, with contents of 0.66 ± 0.06 and 0.70 ± 0.08 mg/g DW, respectively.

Aside from phenolic compounds, caffeine was also quantified in this study (Table 2). High contents of caffeine were widespread in all 30 teas (15.65 ± 1.37 to 47.46 ± 4.35 mg/g DW). The five teas with the highest contents of caffeine were Lushan Yunwu Tea (47.46 ± 4.35 mg/g DW), Duyun Maojian Tea (42.20 ± 3.83 mg/g DW), Yuan’an Luyuan Tea (40.22 ± 3.17 mg/g DW), Weishan Maojian Tea (39.64 ± 2.89 mg/g DW) and Yichang Congou Black Tea (39.55 ± 3.29 mg/g DW), and 5 teas with the lowest contents of caffeine were Lapsang Souchong Black Tea, Wuyi Rock Tea, Tibetan Tea, Qingzhuan Brick Tea, and Tieguanyin Tea.

## 4. Discussion

It is difficult to fully evaluate the antioxidant activities of natural antioxidants just using a single determination method, so employing methods involved in different determination principles are essential [24,25,26]. In this study, 2 commonly used different assays for the determination of antioxidant activities, i.e., FRAP (reducing Fe^3+^ to Fe^2+^) and TEAC (scavenging ATBS radical), were combined to evaluate comprehensively the antioxidant activities of tea infusions [27,28].

The 30 teas possessed potent antioxidant activities (FRAP, 504.80 ± 17.44 to 4647.47 ± 57.87 µmol Fe^2+^/g DW; TEAC, 166.29 ± 24.48 to 2532.41 ± 50.18 µmol Trolox/g DW) and rich phenolic compounds (24.77 ± 2.02 to 252.65 ± 4.74 mg GAE/g DW), which were higher than many other natural products, like 223 medicinal plants (FRAP, 0.14–1844.85 µmol Fe^2+^/g DW; TEAC, 0.99–1544.38 µmol Trolox/g DW; TPC, 0.19–101.33 mg GAE/g DW) [29] and 49 edible macro-fungi (FRAP, 7.905–204.669 µmol Fe^2+^/g DW; TEAC, 4.718–85.719 µmol Trolox/g DW; TPC, 2.440–44.844 mg GAE/g DW) [30]. Among 30 teas, Dianqing Tea, Lushan Yunwu Tea, and Xihu Longjing Tea performed well regarding FRAP, TEAC, and TPC, of which all ranked in the top 5.

The antioxidant capacities and total phenolic contents of 30 teas differed greatly. Cultivar type, place of production, planting condition, harvesting time, leaf grade, and the manufactured process could affect antioxidant capacities and total phenolic contents of teas [11,31]. With respect to manufacturing, the antioxidant properties of teas (obtained from a single cultivar type and planted under the same conditions) could decrease as the fermentation degree increase [32]. Generally speaking, the antioxidant properties of the six categories of teas tested were in decreasing order of green tea, yellow tea, oolong tea, black tea, dark tea, and white tea.

Green tea possessed the highest antioxidant capacity and total phenolic content, which could be due to the minimized oxidation degree of young leaves and the bud due to inactivated enzymes during the steaming process [33]. The antioxidant properties of black and dark teas decreased during extended fermentation by polyphenol oxidases and microbes, respectively [13]. Additionally, the antioxidant activity of white tea, expected to be intermediate between yellow and oolong teas, was similar to fermented teas, which might be related to its special manufacturing process. In the production of white tea, the polyphenol oxidases and peroxidases remain active due to lack of steaming [34]. Furthermore, polyphenols in white tea are gradually lost due to enzymatic and non-enzymatic oxidation reactions in the process of withering.

Phenolic compounds refer to a large class of secondary metabolites in natural products involved in an extensive range of bioactivities ameliorating risks of oxidative stress-related diseases [35,36]. Some phenolic compounds (e.g., tannins) could reduce diet digestibility due to binding and precipitating dietary carbohydrates, proteins, and digestive enzymes, which were considered as antinutrients [37]. From the point of view, malnourished people should drink less tea, and overnourished/obese people could drink more tea. Additionally, some phenolic compounds have several health benefits, like cardiovascular protection and anticancer effects, which are associated with their strong antioxidant activities [3,8]. There were strong correlations between values of FRAP, TEAC and TPC of 30 teas, which indicated that rich phenolic compounds were the main sources responsible for the potent antioxidant activities regarding reducing and free radicals scavenging abilities.

In this study, polyphenols in 30 teas were determined with catechins being the dominant phenolic compounds. Moreover, green tea was a richer source of catechins among 6 categories of teas, in which catechins comprised 11.08%–16.21% of the dry weight. EGCG was the richest catechins. It has been reported that EGCG possessing the most number of phenolic hydroxyl groups manifests the strongest antioxidant activity in catechins, which is even stronger than vitamins C and E [38]. It has been reported that EGCG could exhibit multiple bioactivities, especially anticancer effects, through inhibiting cancer stem cells and modulating molecular events associated with cancer cell proliferation, apoptosis, immunity and so on [39]. Moreover, gallic acid, a well-known phenolic compound, widely presented in all 30 teas in low contents. A study reported that gallic acid could exhibit stronger DPPH radical scavenging capacity than vitamin C [40]. During the process of enzymatic fermentation, catechins in fresh tea leaves could partially convert into the complex condensation product, e.g., theaflavins, driven by polyphenol oxidase-catalyzed oxidative polymerization [41]. In this study, the theaflavin could be detected in two black teas, i.e., Dianhong Congou Black Tea and Yichang Congou Black Tea. A study suggested that theaflavins present in black tea could possess similar levels of antioxidant activities as catechins in green tea in terms of inhibiting Cu^2+^-mediated low-density lipoprotein (LDL) oxidation [42]. Therefore, wide spectra of phenolic compounds were included in the bioactive constituents of tea infusions.

Apart from phenolic compounds, habitual tea infusion intake could also introduce high contents of caffeine making up 1.24%–4.75% of the dry weight of tea leaves. A clinical trial has demonstrated that high caffeine intake could accelerate weight loss via relatively higher thermogenesis and fat oxidation [43,44]. However, the nervous system stimulating effects of caffeine should also be taken into consideration [45].

## 5. Conclusions

In this study, the infusions of 30 selected teas were prepared mimicking daily practices, and their antioxidant activities were evaluated using different determination methods. The results suggested that 30 tea infusions possessed strong antioxidant activities and rich phenolic compounds, which varied greatly in specific varieties. Additionally, phenolic compounds were the main contributors to the antioxidant capacities of teas. Thus, tea is a good dietary source of natural antioxidants, especially phenolic compounds, which have a good potential to be developed into a functional drink or dietary supplements. Furthermore, the antioxidant activities and the profiles of specific bioactive substances (such as EGCG, theaflavin and caffeine) in tea varieties were quite different. Generally speaking, green tea possessed the highest antioxidant capacity and total phenolic content, which was also a richer source of polyphenols, especially catechins. Among 30 teas, Dianqing Tea, Lushan Yunwu Tea, and Xihu Longjing Tea are good sources of natural antioxidants. Thus, consumers should take into account the characteristics of different teas comprehensively for the selection of teas that are best suited to their needs. Moreover, the results are also very helpful for the nutritionist and epidemic experts to calculate or assess the intake amounts of antioxidants from tea.

## Figures and Tables

**Table 1:** The antioxidant capacities and total phenolic contents (TPC) of 30 teas.

No.	Name	Category	Place of Production	FRAP (µmol Fe^2+^/g DW)	TEAC (µmol Trolox/g DW)	TPC (mg GAE/g DW)
1	Dianhong Congou Black Tea	Black Tea	Kunming, Yunnan	2670.13 ± 34.02	994.56 ± 12.64	101.29 ± 1.58
2	Keemun Black Tea	Black Tea	Qimen, Anhui	894.13 ± 20.13	739.88 ± 49.11	62.16 ± 0.99
3	Lapsang Souchong Black Tea	Black Tea	Wuyishan, Fujian	530.13 ± 23.44	471.17 ± 2.02	37.23 ± 0.28
4	Yichang Congou Black Tea	Black Tea	Yichang, Hubei	1039.47 ± 19.73	1034.29 ± 17.99	101.94 ± 4.14
5	Fuzhuan Brick Tea	Dark Tea	Anhua, Hubei	2516.80 ± 76.32	1173.49 ± 12.85	147.11 ± 2.59
6	Liupao Tea	Dark Tea	Wuzhou, Guangxi	1382.13 ± 62.01	932.64 ± 13.27	90.55 ± 2.46
7	Pu-erh Tea	Dark Tea	Pu’er, Yunnan	1787.47 ± 42.39	727.02 ± 33.68	76.08 ± 2.06
8	Qingzhuan Brick tea	Dark Tea	Chibi, Hubei	1170.13 ± 25.72	580.49 ± 5.88	68.65 ± 0.98
9	Tibetan Tea	Dark Tea	Ya’an, Sichuan	504.80 ± 17.44	166.29 ± 24.48	24.77 ± 2.02
10	Dianqing Tea	Green Tea	Kunming, Yunnan	4647.47 ± 57.87	2532.41 ± 50.18	252.65 ± 4.74
11	Dongting Biluochun Tea	Green Tea	Suzhou, Jiangsu	3788.80 ± 80.30	1889.22 ± 12.33	218.38 ± 4.00
12	Duyun Maojian Tea	Green Tea	Duyun, Guizhou	3771.47 ± 25.40	1880.54 ± 45.15	218.55 ± 3.90
13	Enshi Yulu Tea	Green Tea	Enshi, Hubei	3074.13 ± 26.03	1641.77 ± 10.47	174.10 ± 1.88
14	Lu’an Guapian Tea	Green Tea	Lu’an, Anhui	2824.80 ± 107.63	1498.51 ± 17.93	148.16 ± 2.72
15	Lushan Yunwu Tea	Green Tea	Jiujiang, Jiangxi	4099.47 ± 105.10	2353.21 ± 50.68	235.27 ± 7.28
16	Taiping Houkui Tea	Green Tea	Huangshan, Anhui	3250.13 ± 96.03	1662.39 ± 29.54	176.53 ± 4.97
17	Xihu Longjing Tea	Green Tea	Hangzhou, Zhejiang	3872.80 ± 38.16	1935.89 ± 26.32	218.46 ± 8.82
18	Yongxi Huoqing Tea	Green Tea	Jingxian, Anhui	3640.80 ± 89.08	1850.15 ± 17.93	204.32 ± 3.94
19	Fenghuang Shuixian Tea	Oolong Tea	Chao’an, Guangdong	1531.47 ± 38.85	1487.58 ± 21.42	150.10 ± 0.56
20	Luohan Chenxiang Tea	Oolong Tea	Mengdingshan, Sichuan	1055.47 ± 30.02	1207.19 ± 23.34	102.83 ± 2.76
21	Tieguanyin Tea	Oolong Tea	Anxi, Fujian	1567.47 ± 50.01	1084.52 ± 17.64	86.83 ± 0.61
22	Wuyi Rock Tea	Oolong Tea	Wuyishan, Fujian	2002.13 ± 47.72	1065.83 ± 15.80	95.88 ± 2.31
23	Gongmei White Tea	White Tea	Nanping, Fujian	1271.47 ± 29.48	620.10 ± 24.56	75.92 ± 1.46
24	Shoumei White Tea	White Tea	Nanping, Fujian	911.47 ± 44.06	381.3 ± 18.701	51.92 ± 0.56
25	White Peony Tea	White Tea	Nanping, Fujian	1299.47 ± 40.07	904.85 ± 24.58	77.29 ± 2.33
26	Huoshan Large Yellow Tea	Yellow Tea	Lu’an, Anhui	2975.47 ± 85.54	1304.76 ± 29.66	150.99 ± 1.79
27	Junshan Yinzhen Tea	Yellow Tea	Yueyang, Hunan	3292.80 ± 24.33	1589.95 ± 51.41	192.69 ± 0.74
28	Mengding Huangya Tea	Yellow Tea	Mengdingshan, Sichuan	3590.13 ± 54.60	1621.63 ± 7.07	183.31 ± 2.66
29	Weishan Maojian Tea	Yellow Tea	Ningxiang, Hunan	3967.47 ± 87.76	1761.96 ± 30.56	213.05 ± 1.61
30	Yu’an Luyuan Tea	Yellow Tea	Yichang, Hubei	4088.80 ± 118.39	1835.52 ± 19.60	220.08 ± 1.75

FRAP, ferric-reducing antioxidant power; TEAC, Trolox equivalent antioxidant capacity; TPC, total phenolic content.

**Table 2:** The contents of phenolic compounds and caffeine in 30 teas.

**Name**	**Catechin** **(mg/g DW)**	**Epicatechin** **(mg/g DW)**	**Gallocatechin** **(mg/g DW)**	**Epigallocatechin** **(mg/g DW)**	**Catechin gallate** **(mg/g DW)**	**Epicatechin gallate** **(mg/g DW)**	**Gallocatechin Gallate** **(mg/g DW)**		**Epigallocatechin Gallate** **(mg/g DW)**	
Dianhong Congou Black Tea	-	1.33 ± 0.16 ^k,l^	-	-	-	2.72 ± 0.20 ^h,i^	-		-	
Keemun Black Tea	-	0.45 ± 0.04 ^l^	-	-	-	1.12 ± 0.09 ^j^	-		1.34 ± 0.15 ^f^	
Lapsang Souchong Black Tea	-	-	-	-	-	-	-		-	
Yichang Congou Black Tea	-	-	-	-	-	2.69 ± 0.27 ^h,i^	-		2.15 ± 0.15 ^f^	
Fuzhuan Brick Tea	3.22 ± 0.16 ^b^	9.60 ± 0.77 ^b,c^	5.78 ± 0.38 ^e,f,g^	17.24 ± 1.58 ^g,h,i,j^	-	10.92 ± 0.78 ^e^	1.61 ± 0.20 ^i^		9.56 ± 0.44 ^d,e,f^	
Liupao Tea	1.36 ± 0.13 ^c^	3.76 ± 0.29 ^i,j^	3.12 ± 0.27 ^h^	3.70 ± 0.26 ^l^	-	-	-		-	
Pu-erh Tea	-	0.83 ± 0.05 ^k,l^	-	-	-	-	-		-	
Qingzhuan Brick tea	-	3.29 ± 0.20 ^j^	2.48 ± 0.14 ^h^	19.9 ± 1.82 ^g,h,i,j^	-	-	1.46 ± 0.12 ^i^		3.72 ± 0.26 ^f^	
Tibetan Tea	-	-	-	-	-	-	-		0.99 ± 0.09 ^f^	
Dianqing Tea	4.54 ± 0.34 ^a^	5.83 ± 0.56 ^f,g,h^	3.27 ± 0.24 ^h^	13.44 ± 1.07 ^j,k^	0.71 ± 0.10 ^c^	30.29 ± 2.87 ^a^	4.37 ± 0.47 ^g,h^		55.38 ± 5.57 ^a^	
Dongting Biluochun Tea	-	7.05 ± 0.71 ^e,f,g^	5.34 ± 0.40 ^e,f,g^	37.42 ± 2.54 ^e^	0.99 ± 0.08 ^b^	32.12 ± 3.11 ^a^	5.04 ± 0.46 ^f,g^		52.38 ± 4.86 ^a^	
Duyun Maojian Tea	-	9.13 ± 0.65 ^b,c,d^	6.28 ± 0.13 ^d,e,f^	47.86 ± 4.45 ^d^	-	22.56 ± 2.26 ^c,d^	7.24 ± 0.61 ^c,d^		56.12 ± 4.67 ^a^	
Enshi Yulu Tea	3.51 ± 0.26 ^b^	6.98 ± 0.51 ^e,f,g^	5.10 ± 0.12 ^f,g^	34.59 ± 3.05 ^e,f^	1.01 ± 0.08 ^b^	18.5 ± 1.62 ^d^	6.15 ± 0.41 ^c,d,e,f^		35.01 ± 3.16 ^c^	
Lu’an Guapian Tea	-	7.44 ± 0.77 ^d,e,f^	9.78 ± 0.42 ^a^	91.62 ± 6.25 ^a^	-	7.33 ± 0.69 ^e,f,g,h^	5.10 ± 0.45 ^f,g^		34.36 ± 2.56 ^c^	
Lushan Yunwu Tea	-	9.04 ± 0.50 ^b,c,d^	6.37 ± 0.26 ^d,e^	62.56 ± 3.83 ^c^	0.54 ± 0.05 ^c^	19.08 ± 1.46 ^d^	7.00 ± 0.83 ^c,d,e^		57.56 ± 5.54 ^a^	
Taiping Houkui Tea	-	10.59 ± 0.95 ^a,b^	8.88 ± 0.47 ^a,b^	72.73 ± 4.73 ^b^	-	9.03 ± 1.06 ^e,f^	5.44 ± 0.42 ^d,e,f,g^		40.89 ± 3.76 ^c^	
Xihu Longjing Tea	-	7.10 ± 0.26 ^e,f,g^	5.85 ± 0.47 ^e,f,g^	25.58 ± 1.70 ^f,g,h,i^	1.46 ± 0.13 ^a^	24.51 ± 1.85 ^b,c^	9.11 ± 1.10 ^b^		51.85 ± 4.04 ^a,b^	
Yongxi Huoqing Tea	-	8.56 ± 0.46 ^c,d,e^	4.84 ± 0.38 ^g^	21.44 ± 1.62 ^g,h,i,j^	0.53 ± 0.07 ^c^	20.79 ± 1.54 ^c,d^	5.38 ± 0.51 ^e,f,g^		52.77 ± 3.58 ^a^	
Fenghuang Shuixian Tea	-	2.45 ± 0.20 ^j,k^	3.16 ± 0.32 ^h^	16.22 ± 1.06 ^i,j,k^	-	7.57 ± 0.51 ^e,f,g^	3.01 ± 0.31 ^h,i^		34.74 ± 3.17 ^c^	
Luohan Chenxiang Tea	-	5.73 ± 0.54 ^f,g,h^	9.15 ± 0.80 ^a,b^	73.97 ± 7.25 ^b^	-	3.08 ± 0.34 ^g,h,i,j^	2.69 ± 0.21 ^h,i^		17.08 ± 1.62 ^d^	
Tieguanyin Tea	-	9.19 ± 0.87 ^b,c^	7.12 ± 0.53 ^c,d^	71.62 ± 3.77 ^b,c^	-	3.08 ± 0.40 ^g,h,i,j^	1.56 ± 0.17 ^i^		10.34 ± 0.56 ^d,e,f^	
Wuyi Rock Tea	-	3.30 ± 0.22 ^j^	7.91 ± 0.53 ^b,c^	26.28 ± 2.10 ^f,g,h^	-	3.51 ± 0.38 ^g,h,i,j^	2.69 ± 0.23 ^h,i^		14.01 ± 1.06 ^d,e^	
Gongmei White Tea	-	1.07 ± 0.08 ^k,l^	-	6.69 ± 0.59 ^k,l^	-	2.37 ± 0.22 ^i,j^	-		4.93 ± 0.54 ^e,f^	
Shoumei White Tea	-	0.83 ± 0.07 ^k,l^	-	-	-	-	-		2.11 ± 0.13 ^f^	
White Peony Tea	-	1.35 ± 0.13 ^k,l^	-	-	-	2.67 ± 0.22 ^h,i,j^	-		5.35 ± 0.58 ^e,f^	
Huoshan Large Yellow Tea	-	2.44 ± 0.29 ^j,k^	9.76 ± 0.62 ^a^	12.12 ± 0.93 ^j,k,l^	-	6.21 ± 0.53 ^f,g,h,i^	13.10 ± 1.17 ^a^		18.55 ± 2.50 ^d^	
Junshan Yinzhen Tea	0.95 ± 0.06 ^c^	5.46 ± 0.49 ^g,h,i^	3.33 ± 0.32 ^h^	11.96 ± 0.62 ^j,k,l^	-	24.03 ± 2.18 ^b,c^	6.77 ± 0.53 ^c,d,e,f^		42.38 ± 4.32 ^b,c^	
Mengding Huangya Tea	-	5.17 ± 0.56 ^h,i^	3.55 ± 0.16 ^h^	17.07 ± 1.54 ^h,i,j^	1.10 ± 0.09 ^b^	21.99 ± 1.56 ^c,d^	-		37.83 ± 3.06 ^c^	
Weishan Maojian Tea	3.05 ± 0.11 ^b^	12.06 ± 1.17 ^a^	5.03 ± 0.26 ^g^	39.23 ± 2.21 ^d,e^	-	28.68 ± 2.60 ^a,b^	-		38.89 ± 4.16 ^c^	
Yuan’an Luyuan Tea	-	6.53 ± 0.59 ^f,g,h^	5.65 ± 0.40 ^e,f,g^	26.61 ± 1.25 ^f,g^	-	21.38 ± 1.53 ^c,d^	7.32 ± 0.91 ^c^		54.38 ± 4.73 ^a^	
**Name**	**Gallic Acid** **(mg/g DW)**	**Ellagic Acid** **(mg/g DW)**	**Quercitrin** **(mg/g DW)**	**Astragalin** **(mg/g DW)**	**Quercetin** **(mg/g DW)**	**Kaempferol** **(mg/g DW)**	**Chlorogenic Acid (mg/g DW)**	**Myricetin** **(mg/g DW)**	**Caffeine** **(mg/g DW)**	**Theaflavin** **(mg/g DW)**
Dianhong Congou Black Tea	2.65 ± 0.17 ^c,d^	4.57 ± 0.41 ^c,d^	-	1.09 ± 0.16 ^c^	-	-	-	-	35.09 ± 3.62 ^b,c,d,e,f,g,h^	0.66 ± 0.06 ^a^
Keemun Black Tea	2.87 ± 0.15 ^b,c,d^	-	0.65 ± 0.07 ^i^	0.89 ± 0.05 ^c,d,e^	-	-	-	-	25.07 ± 2.86 ^i,j,k^	-
Lapsang Souchong Black Tea	1.88 ± 0.08 ^f,g,h^	2.11 ± 0.27 ^h^	0.81 ± 0.09 ^g,h,i^	0.60 ± 0.04 ^d,e,f^	-	-	-	-	21.09 ± 2.09 ^j,k,l,m^	-
Yichang Congou Black Tea	3.77 ± 0.32 ^a^	2.78 ± 0.24 ^e,f,g,h^	-	3.54 ± 0.42 ^a^	-	-	-	-	39.55 ± 3.29 ^a,b,c,d,e^	0.70 ± 0.08 ^a^
Fuzhuan Brick Tea	3.22 ± 0.22 ^b^	2.51 ± 0.14 ^e,f,g,h^	1.86 ± 0.19 ^b^	1.75 ± 0.16 ^b^	0.08 ± 0.01 ^a^	0.19 ± 0.02 ^a^	0.35 ± 0.02 ^b,c^	-	27.8 ± 2.29 ^h,i,j^	-
Liupao Tea	2.10 ± 0.19 ^f,g^	2.31 ± 0.27 ^f,g,h^	0.79 ± 0.04 ^g,h,i^	-	-	-	-	-	30.98 ± 2.79 ^e,f,g,h,i^	-
Pu-erh Tea	1.23 ± 0.02 ^k,l,m^	2.61 ± 0.19 ^e,f,g,h^	0.74 ± 0.03 ^h,i^	-	-	-	-	-	26.58 ± 1.30 ^i,j,k^	-
Qingzhuan Brick tea	2.85 ± 0.20 ^b,c,d^	-	-	-	-	-	-	-	15.65 ± 1.37 ^m,n^	-
Tibetan Tea	1.52 ± 0.12 ^h,i,j,k,l^	-	-	-	-	-	-	-	16.32 ± 1.31 ^l,m,n^	-
Dianqing Tea	1.77 ± 0.13 ^g,h,i^	7.77 ± 0.70 ^b^	-	-	-	-	0.49 ± 0.02 ^a^	-	38.01 ± 2.46 ^b,c,d,e,f^	-
Dongting Biluochun Tea	1.03 ± 0.04 ^m^	-	1.15 ± 0.11 ^d,e,f,g^	1.02 ± 0.11 ^c,d^	-	-	0.29 ± 0.01 ^c,d,e^	-	32.45 ± 2.53 ^c,d,e,f,g,h,i^	-
Duyun Maojian Tea	1.32 ± 0.08 ^j,k,l,m^	3.50 ± 0.26 ^d,e^	2.53 ± 0.19 ^a^	-	-	-	-	-	42.20 ± 3.83 ^a,b^	-
Enshi Yulu Tea	1.80 ± 0.06 ^f,g,h^	3.52 ± 0.29 ^d,e^	1.08 ± 0.07 ^d,e,f,g,h^	-	-	-	-	-	38.14 ± 3.01 ^b,c,d,e,f^	-
Lu’an Guapian Tea	0.45 ± 0.02 ^n^	-	1.01 ± 0.11 ^e,f,g,h,i^	-	-	-	0.33 ± 0.02 ^b,c^	-	28.74 ± 2.62 ^g,h,i,j^	-
Lushan Yunwu Tea	1.24 ± 0.10 ^k,l,m^	3.12 ± 0.30 ^e,f,g,h^	2.76 ± 0.25 ^a^	-	-	-	-	-	47.46 ± 4.35 ^a^	-
Taiping Houkui Tea	0.58 ± 0.07 ^n^	2.33 ± 0.22 ^f,g,h^	1.22 ± 0.15 ^d,e,f^	0.39 ± 0.02 ^f^	-	-	0.35 ± 0.03 ^b,c^	-	32.37 ± 3.17 ^c,d,e,f,g,h,i^	-
Xihu Longjing Tea	1.63 ± 0.04 ^h,i,j,k^	-	-	-	-	-	0.39 ± 0.03 ^b^	-	36.65 ± 3.28 ^b,c,d,e,f,g^	-
Yongxi Huoqing Tea	1.36 ± 0.03 ^i,j,k,l,m^	3.44 ± 0.20 ^d,e,f^	-	-	-	-	0.34 ± 0.03 ^b,c^	-	31.77 ± 2.50 ^c,d,e,f,g,h,i^	-
Fenghuang Shuixian Tea	3.05 ± 0.14 ^b,c^	2.29 ± 0.16 ^g,h^	1.65 ± 0.12 ^b,c^	1.22 ± 0.06 ^c^	-	-	0.24 ± 0.01 ^d,e^	-	31.66 ± 1.97 ^d,e,f,g,h,i^	-
Luohan Chenxiang Tea	0.59 ± 0.02 ^n^	-	1.28 ± 0.13 ^c,d,e^	0.63 ± 0.07 ^d,e,f^	-	-	0.30 ± 0.02 ^c,d^	-	24.88 ± 1.65 ^i,j,k^	-
Tieguanyin Tea	0.29 ± 0.02 ^n^	2.15 ± 0.20 ^h^	0.95 ± 0.05 ^e,f,g,h,i^	-	-	-	0.23 ± 0.02 ^e^	-	12.36 ± 1.18 ^n^	-
Wuyi Rock Tea	1.35 ± 0.08 ^i,j,k,l,m^	2.10 ± 0.20 ^h^	-	-	-	-	-	-	19.28 ± 1.26 ^k,l,m,n^	-
Gongmei White Tea	2.57 ± 0.02 ^d,e^	2.30 ± 0.21 ^g,h^	0.89 ± 0.05 ^f,g,h,i^	-	-	-	-	-	27.54 ± 1.83 ^h,i,j,k^	-
Shoumei White Tea	2.09 ± 0.07 ^f,g^	-	-	0.36 ± 0.05 ^f^	-	-	-	-	24.52 ± 2.18 ^i,j,k,l^	-
White Peony Tea	2.71 ± 0.10 ^c,d^	2.35 ± 0.10 ^f,g,h^	0.94 ± 0.05 ^e,f,g,h,i^	0.50 ± 0.05 ^e,f^	-	0.17 ± 0.02 ^a^	0.26 ± 0.02 ^d,e^	-	30.64 ± 2.92 ^f,g,h,i^	-
Huoshan Large Yellow Tea	3.16 ± 0.26 ^b^	2.39 ± 0.25 ^e,f,g,h^	0.89 ± 0.08 ^f,g,h,i^	-	0.01 ± 0.00 ^b^	0.19 ± 0.01 ^a^	-	-	32.51 ± 2.20 ^c,d,e,f,g,h,i^	-
Junshan Yinzhen Tea	1.19 ± 0.06 ^l,m^	3.29 ± 0.13 ^e,f,g^	1.43 ± 0.14 ^c,d^	-	-	-	0.35 ± 0.02 ^b,c^	-	38.66 ± 2.50 ^b,c,d,e,f^	-
Mengding Huangya Tea	2.22 ± 0.17 ^e,f^	9.79 ± 1.01 ^a^	-	-	-	-	-	-	35.61 ± 3.25 ^b,c,d,e,f,g,h^	-
Weishan Maojian Tea	1.22 ± 0.04 ^k,l,m^	4.48 ± 0.45 ^c,d^	-	-	-	-	0.33 ± 0.01 ^b,c^	-	39.64 ± 2.89 ^a,b,c,d^	-
Yu’an Luyuan Tea	1.73 ± 0.16 ^g,h,i,j^	4.77 ± 0.44 ^c^	-	-	-	-	0.34 ± 0.03 ^b,c^	-	40.22 ± 3.17 ^a,b,c^	-

Different superscript lowercase letters within a column indicate statistical significance at *p* < 0.05.
